# Impact on clinical practice of the implementation of guidelines for the toxicity management of targeted therapies in kidney cancer. The protect-2 study

**DOI:** 10.1186/s12885-016-2084-9

**Published:** 2016-02-22

**Authors:** Nuria Lainez, Jesús García-Donas, Emilio Esteban, Javier Puente, M. Isabel Sáez, Enrique Gallardo, Álvaro Pinto-Marín, Sergio Vázquez-Estévez, Luis León, Icíar García-Carbonero, Cristina Suárez-Rodríguez, Carmen Molins, Miguel A. Climent-Duran, Martín Lázaro-Quintela, Aranzazu González del Alba, María José Méndez-Vidal, Isabel Chirivella, Francisco J. Afonso, Marta López-Brea, Nuria Sala-González, Montserrat Domenech, Laura Basterretxea, Carmen Santander-Lobera, Irene Gil-Arnáiz, Ovidio Fernández, Cristina Caballero-Díaz, Begoña Mellado, David Marrupe, José García-Sánchez, Ricardo Sánchez-Escribano, Eva Fernández Parra, José C. Villa Guzmán, Esther Martínez-Ortega, María Belén González, Marina Morán, Beatriz Suarez-Paniagua, María J. Lecumberri, Daniel Castellano

**Affiliations:** Department of oncology, Complejo Hospitalario de Navarra, Servicio Oncología Médica. Pabellón B 2ª planta. Hospital de día, C/ Irunlarrea, 3, 31008 Pamplona, Navarra Spain; Department of oncology, Hospital Sanchinarro, C/ Oña, 10, 28050 Madrid, Spain; Department of oncology, Hospital Universitario Central de Asturias, Julián Clavería s/n, 33006 Oviedo, Spain; Department of oncology, Hospital Clínico de Madrid, C/ Doctor Martín Lagos s/n, 28040 Madrid, Spain; Department of oncology, Hospital Universitario Clínico Virgen de la Victoria, Campus Universitario de Teatinos, s/n, 29010 Málaga, Spain; Department of oncology, Parc Taulí Sabadell Hospital Universitari, Parc Taulí 1, 08208 Sabadell, Spain; Department of oncology, Hospital Universitario La Paz, P de la Castellana 261, 28046 Madrid, Spain; Department of oncology, Hospital Universitario Lucus Augusti, Lugar San Cibrao, S/N, 27003 Lugo, Spain; Department of oncology, Hospital Santiago de Compostela, Travesía da Choupana, s/n, 15706 Santiago de Compostela, Spain; Department of oncology, Hospital Virgen de la Salud, Adva. De Barber, 30, 45004 Toledo, Spain; Department of oncology, Hospital Vall d’Hebron, Ps Vall d’Hebron, 119-129, 8035 Barcelona, Spain; Department of oncology, Hospital Universitario Dr. Peset, Avda. Gaspar Aguilar 90, 46017 Valencia, Spain; Department of oncology, Instituto Valenciano de Oncología, Gregorio Gea, 31-1° Planta, 46009 Valencia, Spain; Department of oncology, Complexo Hospitalario Universitario de Vigo, Pizarro 22, 36204 Vigo, Spain; Department of oncology, Hospital Universitario Son Espases, Ctra Valldemossa, 79, 07010 Palma de Mallorca, Spain; Department of oncology, Hospital Universitario Reina Sofía, Avda. Menéndez Pidal, s/n, 14004 Córdoba, Spain; Department of oncology, Hospital Clínico de Valencia, Avda Blasco Ibáñez, 17, 46010 Valencia, Spain; Department of oncology, Complejo Hospitalario Arquitecto Marcide, Rúa da residencia s/n. San pedro de Leixa, 15405 Ferrol, Spain; Department of oncology, Hospital Marqués de Valdecilla, Avda. Valdecila s/n, 39008 Santander, Spain; Department of oncology, ICO de Girona, Francia s/n, 17007 Gerona, Spain; Department of oncology, Hospital de Althaia Xarxa Asistencial Manresa, Dr. Joan Soler, 1-3, 08243 Barcelona, Spain; Department of oncology, Hospital de Donostia, P° Dr. Beguiristain 109, 20014 San Sebastian, Spain; Department of oncology, Hospital Miguel Servet, Avda Gómez Laguna 25, 50009 Zaragoza, Spain; Department of oncology, Hospital Reina Sofía, Carretera Tarazona, KM 3, 31500 Tudela, Spain; Department of oncology, Complejo Hospitalario Ourense. Hospital Santa María Nai, Ramón Puga, 52-54, 32005 Orense, Spain; Department of oncology, Hospital General Universitario de Valencia, Avda Tres Cruces, s/n, 46014 Valencia, Spain; Department of oncology, IDIBAPS, Hospital Clinic i Provincial de Barcelona, Villarroel, 170, 08036 Barcelona, Spain; Department of oncology, Hospital Universitario de Móstoles, Río Júcar s/n, 28935 Móstoles, Madrid Spain; Department of oncology, Hospital Arnau de Vilanova, C/ San Clemente n 12, 46015 Valencia, Spain; Department of oncology, Hospital Universitario de Burgos, Avenida Cid Campeador, 96, 09005 Burgos, Spain; Department of oncology, H.U. Hospital de Valme, Ctra. de Cádiz Km. 548.9, 41014 Sevilla, Spain; Department of oncology, Hospital de Ciudad Real, Obispo Rafael Torija, 13005 Ciudad Real, Spain; Department of oncology, Hospital Ciudad de Jaén, Avenida Ejercito Español 10, 23007 Jaén, Spain; Department of oncology, Hospital Son Llatzer, Ctra. Manacor, km.4, Sont Frriol, 07198 Palma de Mallorca, Spain; Pfizer Madrid, Avda. de Europa, 20B, 28108 Alcobendas, Madrid Spain; Trial Form Support, Avda. de Europa, 20B, 28108 Alcobendas, Madrid Spain; Department of oncology, Hospital 12 de Octubre, Av. de Córdoba s/n, 28041 Madrid Spain

**Keywords:** Adverse events, Guidelines, Renal cell carcinoma, Targeted therapy

## Abstract

**Background:**

The impact of such recommendations after their implementation of guidelines has not usually been evaluated. Herein, we assessed the impact and compliance with the Spanish Oncology Genitourinary Group (SOGUG) Guidelines for toxicity management of targeted therapies in metastatic renal cell carcinoma (mRCC) in daily clinical practice.

**Methods:**

Data on 407 mRCC patients who initiated first-line targeted therapy during the year before and the year after publication and implementation of the SOGUG guideline program were available from 34 Spanish Hospitals. Adherence to SOGUG Guidelines was assessed in every cycle.

**Results:**

Adverse event (AE) management was consistent with the Guidelines as a whole for 28.7 % out of 966 post-implementation cycles compared with 23.1 % out of 892 pre-implementation cycles (*p* = 0.006). Analysis of adherence by AE in non-compliant cycles showed significant changes in appropriate management of hypertension (33 % pre-implementation vs. 44.5 % post-implementation cycles; *p* < 0.0001), diarrhea (74.0 % vs. 80.5 %; *p* = 0.011) and dyslipemia (25.0 % vs. 44.6 %; *p* < 0.001).

**Conclusions:**

Slight but significant improvements in AE management were detected following the implementation of SOGUG recommendations. However, room for improvement in the management of AEs due to targeted agents still remains and could be the focus for further programs in this direction.

## Background

Targeted therapies have led to clinically meaningful advances in the treatment of patients with metastatic renal cell carcinoma (mRCC).

Different antiangiogenic agents targeting different various steps along the angiogenesis pathway, inhibiting tumor growth and new vessel growth are available. Bevacizumab is a monoclonal antibody against VEGF-A [1]. Pazopanib is a highly potent tyrosine kinase inhibitor (TKI) that targets vascular endothelial growth factor receptors (VEGFR) − 1, −2 and −3, platelet-derived growth factor receptor (PDGFR) − α and β and c-Kit [2]. Sorafenib is a multi-targeted kinase inhibitor that targets RAF kinases (CRAF, BRAF, V600 BRAF) and tyrosine kinases receptor (the stem cell factor c-KIT, fetal liver tyrosine kinase 3 (FLT-3), VEGFR-2, VEGFR-3, and PDGFR-β) [3]. Sunitinib inhibits PDGFR-α, PDGFR-β, VEGFR-1, VEGFR-2, VEGFR-3, cKIT, FLT3, Colony-stimulating factor 1 receptor (CSF-1R) and the Glial cell line-derived neurotrophic factor receptor [4–6]. Finally, both approved mammalian targets of rapamycin (mTOR inhibitors), temsirolimus and everolimus, are derivatives of the natural compound rapamycin. To inhibit mTOR signaling, temsirolimus and everolimus interact with the cytosolic FK506-binding protein- 12 (FKBP12) to form a complex which binds the mTOR. Through their effects on mTOR, these drugs can inhibit cell proliferation and induce apoptosis, in addition to the inhibiton of angiogenesis [7, 8].

These novel antiangiogenic agents have different mechanisms of action and exhibit a distinct toxicity profile that requires appropriate monitoring and management. Commonly reported toxicities for antiangiogenic agents include hypertension, skin reactions, asthenia, fatigue, gastrointestinal disturbances, hepatotoxicity, metabolic dysfunctions and pneumonitis [9, 10]. Adverse Event (AE) management is a critical component of the overall care of patients with mRCC [11]. Subanalyses of clinical trials in mRCC have concluded that some AEs induced by these therapies may be associated with a better outcome [12–14]. Thus, appropriate management of adverse effects seems to be key in order to maintain optimal doses in those patients who could obtain a major benefit from treatment.

The use of valid guidelines can improve clinical practice, especially if accompanied by effective dissemination strategies. However, both the context within which guidelines are delivered and the nature of targeted clinical behaviors may also influence their uptake. With the aim of improving the AE management of targeted therapies, the Spanish Oncology Genitourinary Group (SOGUG) published in 2011[15] a Guide of recommendations for AE management and launched a program for the diffusion and implementation of this guide. In this study we have evaluated the impact and compliance with this Guide in the daily clinical practice.

## Methods

The Guidelines for the management of side effects of targeted therapies were designed by the “Toxicity, Rare Tumors and Hereditary Cancer Working Group” of the SOGUG. They were published in March 2011 and distributed in PDF and paper format among all SOGUG members (245 Medical oncologists from 118 institutions). Additionally, free copies were available for attendees at several national meetings on genitourinary tumors and became publically available through a web application (http://www.sogug.es/Assets/docs/manejo_farmacos_antidiana_cancer_renal.pdf).

For the implementation of the Guidelines 12 oncologists from the above mentioned working group were specifically trained on the recommendations provided by the guides. Nine meetings all around the country were held where clinical cases were presented by local oncologists and discussed with one of the trained oncologists. In total, 120 oncologists became involved in the educational program.

**Fig. 1:**
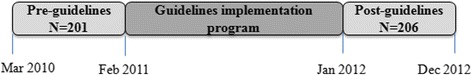
Patient distribution: Patients were recruited during the year before (between March 2010 and February 2011; pre-guidelines population) or the year after (between January 2012 and December 2012; post-guideline population) the publication, diffusion and implementation of the SOGUG Guideline program

Medical records were reviewed of adult patients with histologically confirmed mRCC, who initiated any targeted therapy (sunitinib, sorafenib, pazopanib, everolimus, temsirolimus or bevacizumab) during the year before (between March 2010 and February 2011; pre-guidelines population) or the year after (between January 2012 and December 2012; post-guideline population) of publication, diffusion and implementation of the SOGUG Guideline program (Fig. 1). Demographic, clinical and treatment data including tests performed as screening or monitoring of AEs were collected.

The main AEs related to the different treatment options were registered (Table 1) Hospital category was defined by number of cases diagnosed with renal cancer per year (c/y): primary hospital (≥ 20 c/y); secondary hospital (11–19) c/y and tertiary hospital (0 to 10 c/y) was also recorded.

**Table 1:**
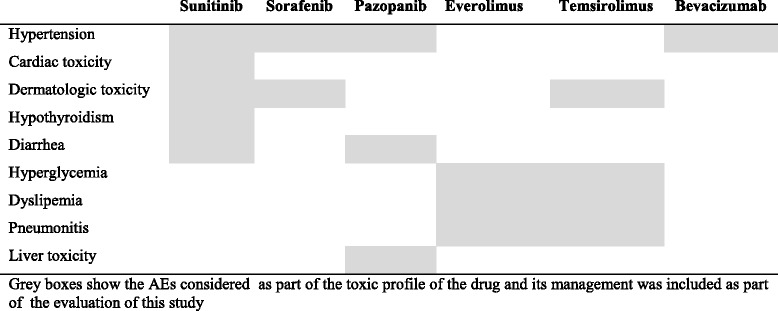
Management of adverse events assessed according to targeted treatment

Patients provided their written informed consent to collect their data. This study was approved by the Spanish Medicines Agency and by the Ethics and Clinical Research Committee of Hospital of Navarra.

Non-compliance criteria with SOGUG Guidelines were defined as: *Hypertension*: Blood pressure level was not determined prior to start of treatment and in every cycle. Perform dose reduction, dose interruption or treatment discontinuation when the blood pressure value was lower than 200/110 mmHg. *Cardiac toxicity:* Basal and three-monthly assessments of left ventricular ejection fraction (LVEF) were not performed. Perform dose reduction or dose interruption due to toxicity grade 1 or treatment discontinuation due to toxicity < 4. *Dermatologic toxicity:* Information about suffering from rash or hand-foot syndrome was not gathered from the first cycle. Perform dose reduction or dose interruption with toxicity of grade < 2. *Hypothyroidism:* Thyroid-stimulating hormone (TSH) level was not determined prior to treatment start and every three months. Carry out dose interruption or treatment discontinuation due to TSH levels. *Hyperglycemia:* Glucose level assessment in every cycle was not performed. *Dyslipemia*: Cholesterol, low density lipoprotein (LDL) and triglyceride levels were not measured from the first cycle. *Diarrhea:* Information about the development of diarrhea was not gathered in all cycles. Carry out dose reduction or dose interruption due to diarrhea grade <3. *Pneumonitis:* Basal chest X-rays, pulmonary function and diffusing capacity of the lungs for carbon monoxide (DLCO) assessments were not performed. Clinical symptoms were not recorded from the first cycle. Patients with positive clinical symptoms were not subjected to chest X-rays and peak expiratory flow (PEF) assessment. Carry out dose reduction due to pneumonitis grade < 3, dose interruption due to pneumonitis grade < 2 or treatment discontinuation due to pneumonitis grade < 4. *Hepatic toxicity:* Liver function tests were not performed prior to start of treatment and at every cycle. Patients with ALT increase between 3 and 8 times the upper limits of normal (ULN) and bilirubin normal value were not subjected to weekly blood test. Carry out dose reduction or dose interruption with ALT < 8 times ULNs value or treatment discontinuation with ALT < 3 times ULN value and bilirubin <2 times ULN value. *Proteinuria:* Clinical information on proteinuria from the first cycle of treatment was not recorded. Carry out dose reduction or dose interruption due to proteinuria grade < 2 or treatment discontinuation due to proteinuria grade <3.

### Statistical analysis

The primary objective was to assess the SOGUG Guidelines compliance before and after their publication and implementation. Secondary objectives included treatment modifications due to Guideline compliance and adherence to the SOGUG recommendations according to the hospital category.

Adherence to SOGUG Guidelines was assessed in every cycle by evaluation of management of the pre-specified AEs according to SOGUG Guideline recommendations [15] (Table 1). AEs were recorded and rated by an external data monitor according to National Cancer Institute Common Terminology Criteria for adverse events (NCI CTCAE) version 4.0.

Student’s *t*-test or Mann-Withney *U* test were used to compare quantitative variables and Pearson’s chi-square test or Fisher’s exact test for qualitative variables. Tests were two-tailed with a significance level of 5 %. Data were analysed using SPSS statistical software v17.0.

## Results

Thirty-four of the 40 institutions of SOGUG finally participated in this retrospective, cross-sectional, multicentre study. The analysis was conducted on 407 out of 410 mRCC patients (201 (49.4 %) pre-implementation, 206 (50.6 %) post-implementation). 1858 of 2103 treatment cycles were deemed as evaluable (892 (48.0 %) pre-implementation, 966 (52.0 %) post-implementation). Most of the non-evaluable cycles were excluded because they had not been administered within the pre-specified timeframe. Table 2 shows patient characteristics. Proportion of men/women and ECOG performance status were similar between pre- and post-implementation groups (p > 0.05). Statistically significant differences were observed regarding the age of patients (median age: 60.5 years, 95 % IC: 58.4 to 61.8 vs. 64.5 years, 95 % IC 62.1 to 65.3; *p* = 0.003) in the pre-implementation and post-implementation groups respectively.

**Table 2:** Patients characteristics

	Total (*N* = 407)
Sex, *n* (%)	
Female	110 (27.0)
Male	297 (73.0)
Mean age (SD), years	61.9 (12.0)
ECOG PS, *n* (%)	370
0	110 (29.7)
1	206 (55.7)
2	44 (11.9)
3	10 (2.7)
Histology, *n* (%)	399
Clear cell	357 (89.5)
Papillary	21 (5.3)
Cromophobe	9 (2.3)
Sarcomatoid	4 (1.0)
Mixed	3 (0.8)
Collecting Duct	1 (0.3)
Others	4 (1.0)
*Targeted treatment, *n* (%)	
Sunitinib	251 (61.7)
Sorafenib	62 (15.2)
Pazopanib	56 (13.8)
Everolimus	70 (17.2)
Temsirolimus	37 (9.1)
Bevacizumab	5 (1.2)

* Some patients received more than one treatment

Cycle distribution and adherence to SOGUG Guidelines according to type of treatment are summarized in Table 3. Overall, compliance with the SOGUG Guidelines was significantly greater in the post-implementation cycles compared with those of the pre-implementation period (28.7 vs. 23.1 %; *p* = 0.006). A meaningful increase of adherence to the Guideline after the training program was observed with everolimus treatment (32.3 % vs. 46.2 % *p* = 0.019), while this did not occur with sunitinib or with temsirolimus treatments, where only a numerical but not a significant improvement was observed. Sorafenib showed a significant decrease in compliance with the guidelines (10.8 % vs. 2.2 %; *p* = 0.013). Pazopanib comparative analysis was not carried out due to the low number of patients included in the pre-implementation group.

**Table 3:** SOGUG Guideline compliance according to treatment

	Sunitinib 977		Sorafenib 247		Pazopanib 210		Everolimus 166		Temsirolimus 125		Bevacizumab 24		Total 1,858	
	*Pre* * 534*	*Post* * 443*	*Pre* * 157*	*Post* * 90*	*Pre* * 1*	*Post* * 209*	*Pre* * 130*	*Post* * 145*	*Pre* * 56*	*Post* * 69*	*Pre* * 14*	*Post* * 10*	*Pre* * 892*	*Post* * 966*
Overall compliance, *n* cycles (%)														
*Yes*	136 (25.5)	123 (27.8)	17 (10.8)	2 (2.2)*	0 (0.0)	68 (32.5)	42 (32.3)	67 (46.2)*	8 (14.3)	17 (24.6)	3 (21.4)	0 (0.0)	206 (23.1)	277 (28.7)*
*No*	398 (75.5)	320 (72.2)	140 (89.2)	88 (97.8)	1 (100)	141 (67.5)	88 (67.7)	78 (53.8)	48 (85.7)	52 (75.4)	11 (78.6)	10 (100)	686 (76.9)	689 (71.3)
Guidelines compliance by adverse event, n cycles (%)^a^														
*Hypertension*	173 (43.5)	171 (53.4)*	1 (0.7)	0 (0.0)	0 (0.0)	69 (48.9)	–	–	–	–	9 (81.8)	9 (90.0)	183 (33.3)	249 (44.5)^£^
*Cardiac toxicity*	113 (28.4)	87 (27.2)	–	–	–	–	–	–	–	–	–	–	113 (28.4)	87 (27.2)
*Skin toxicity*	398 (100)	320 (100)	140 (100)	88 (100)	–	–	–	–	48 (100)	52 (100)-	–	–	586 (100)	460 (100)
*Hypothyroidism*	172 (43.2)	157 (49.1)	–	–	–	–	–	–	–	–	–	–	172 (43.2)	157 (49.1)
*Diarrhea*	274 (68.8)	264 (82.5)^£^	124 (88.6)	73 (83.0)	1 (100)	105 (74.5)	–	–	–	–	–	–	399 (74.0)	442 (80.5)*
*Hyperglycemia*	–	–	–	–	–	–	63 (71.6)	53 (67.9)	28 (58.3)	39 (75.0)	–	–	91 (66.9)	92 (70.8)
*Dyslipemia*	–	–	–	–	–	–	22 (25.0)	42 (53.8) ^£^	12 (25.0)	16 (30.8)	–	–	34 (25.0)	58 (44.6)^#^
*Pneumonitis*	–	–	–	–	–	–	53 (60.2)	42 (53.8)	31 (64.6)	32 (61.5)	–	–	84 (61.8)	74 (56.9)
*Liver toxicity*	–	–	–	–	1 (100)	37 (26.2)	–	–	–	–	–	–	1 (100)	37 (26.2)
*Proteinuria*	–	–	–	–	–	–	–	–	–	–	2 (18.2)	0 (0.0)	2 (18.2)	0 (0.0)

^a^ (%): percentage of compliance in relation to the total cycles in which the SOGUG guidelines were not-complied with

*p between groups <0.05; ^#^ *p* between groups <0.001; ^£^ *p* between groups <0.0001. Length of cycles according to routine clinical practice: sunitinib 6 weeks; other treatments 4 weeks

SOGUG recommendations were not fulfilled as a whole in 71 % of cycles (Table 3). However, when the management of each type of AE in those cycles was analyzed, an improvement was observed in the management of some AEs. Overall, significant increase in the appropriate management of hypertension (pre-implementation 33 % vs. 44.5 % post-implementation; *p* < 0.0001), diarrhea (74.0 % vs. 80.5 %; *p* = 0.011) and dyslipemia (25.0 % vs. 44.6 %; *p* < 0.001) was observed in those cycles where SOGUG recommendations were not fulfilled as a whole (Table 3). In addition, two agents showed significant increase in guideline compliance in some AEs: sunitinib in the management of hypertension (43.5 % vs. 53.4; *p* = 0.008) and diarrhea (68.8 vs. 82.5; *p* < 0.0001) and everolimus in the management of dyslipemia (25.0 % vs. 53.8 %; *p* < 0.0001; Table 3).

The most frequent reason for non-compliance with the Guidelines was the lack of test performing (Table 4): basal and follow-up assessments of blood pressure, LVEF, TSH glucose, chest X-rays, pulmonary function, DLCO and liver function were not performed as frequently as recommended by the Guidelines. Inappropriate dose reductions, interruptions or treatment discontinuation were not reasons for non-compliance with Guidelines in the vast majority of non-compliant cycles (Table 4).

**Table 4:** Reasons for non-compliance with SOGUG guidelines

	Sunitinib *718*	Sorafenib *227*	Pazopanib *142*	Everolimus *166*	Temsirolimus *100*	Bevacizumab *21*
*Hypertension*, *n cycles (%)*	374	227	73	–	–	3
Basal BP not recorded	–	226 (99.6)	27 (37.0)	–	–	3 (100)
BP not recorded	363 (97.1)	137 (60.4)	45 (61.6)	–	–	–
Dose reduction	9 (2.4)	–	1 (1.4)	–	–	–
Dose interruption	1 (0.3)	–	–	–	–	–
Treatment discontinuation	2 (0.5)	1 (0.4)	–	–	–	–
*Cardiac toxicity, n cycles (%)*	518	–	–	–	–	–
Non-recorded basal LVEF	145 (28.0)	–	–	–	–	–
LVEF not performed	484 (93.4)	–	–	–	–	–
Dose reduction	1 (0.2)	–	–	–	–	–
Dose interruption	1 (0.2)	–	–	–	–	–
Treatment discontinuation	2 (0.4)	–	–	–	–	–
*Hypotyroidism, n cycles (%)*	389	–	–	–	–	–
Basal TSH not recorded	114 (29.3)	–	–	–	–	–
TSH > 10 mU/l not-performed	358 (92.0)	–	–	–	–	–
Dose interruption	2 (0.5)	–	–	–	–	–
*Diarrhea, n cycles (%)*	180	31	36	–	–	–
Not recorded	165 (91.7)	29 (93.5)	36 (100)	–	–	–
Dose reduction	12 (6.7)	1 (3.2)	–	–	–	–
Dose interruption	3 (1.7)	1 (3.2)	–	–	–	–
*Hyperglycemia, n cycles (%)*	–	–	–	50	33	–
Not recorded	–	–	–	25 (100)	33 (100)	–
*Dyslipemia, n cycles (%)*	–	–	–	102	72	–
Not recorded	–	–	–	102 (100)	72 (100)	–
*Pneumonitis, n cycles (%)*	–	–	–	71	37	–
Basal data not recorded	–	–	–	66 (93.0)	35 (94.6)	–
Follow-up data not recorded	–	–	–	5 (7.0)	1 (2.7)	–
Treatment discontinuation	–	–	–	2 (2.8)	1 (2.7)	–
*Liver toxicity, n cycles (%)*	–	–	104	–	–	–
Basal liver function not recorded	–	–	19 (18.3)	–	–	–
Liver function not recorded	–	–	81 (77.9)	–	–	–
ALT increase > 3–8 ULN	–	–	5 (4.8)	–	–	–
Dose interruption	–	–	2 (1.9)	–	–	–
Treatment discontinuation	–	–	1 (1.0)	–	–	–
*Proteinuria, n cycles (%)*						
Not performed	–	–	–	–	–	19 (100)

Length of cycles according to routine clinical practice: sunitinib 6 weeks; other treatments 4 weeks

Overall, patients from pre- and post-implementation groups received a median (Q1–Q3) of 4.0 (2.0–6.0) and 4.0 (3.0–6.0) cycles, respectively. Table 5 shows the number of cycles administered according to the targeted agent. In all, 48 (11.8 %) patients needed dose reductions, 33 (8.1 %) dose interruptions, 24 (5.9 %) treatment discontinuation and 4 (1.0 %) dose increases. No statistically significant differences were observed between pre and post-implementation groups for any treatment action taken or targeted agent (Table 5). With regard to the total 1858 cycles, in 58 (3.1 %) of them a dose reduction was carried out, in 38 (2.0 %) a dose interruption, in 26 (1.4 %) a treatment discontinuation and in 4 (0.2 %) an increase of dose. No significant differences after the implementation program were observed for any treatment either.

**Table 5:** Changes in treatment pattern by patients

	Sunitinib 251		Sorafenib 62		Pazopanib 56		Everolimus 70		Temsirolimus 37		Bevacizumab 5	
	*Pre * *143*	*Post * *108*	*Pre* * 43*	*Post * *19*	*Pre * *1*	*Post * *55*	*Pre* * 34*	*Post * *36*	*Pre * *19*	*Post* * 18*	*Pre * *4*	*Post * *1*
Number of cycles administered												
*Median (Q1–Q3)*	4.0 (2.0–5.0)	4.0 (2.0–5.0)	3.0 (2.0–5.0)	3.0 (2.0–8.0)	1.0 (1.0–1.0)	3.0 (2.0–5.0)	3.0 (2.0–5.0)	4.0 (2.0–6.0)	2.0 (1.0–4.0)	2.5 (2.0–4.0)	3.5 (2.5–4.5)	10.0 (10.0–10.0)
*Treatment modification, n cycles (%)												
*Dose reduction*	24 (16.8)	16 (14.8)	4 (9.3)	1 (5.3)	0 (0.0)	4 (7.3)	1 (2.9)	0 (0.0)	0 (0.0)	0 (0.0)	0 (0.0)	0 (0.0)
*Dose interruption*	8 (5.6)	9 (8.3)	2 (4.7)	2 (10.5)	0 (0.0)	4 (7.3)	3 (8.8)	4 (11.1)	1 (5.3)	1 (5.6)	0 (0.0)	0 (0.0)
*Treatment discontinuation*	6 (4.2)	5 (4.6)	3 (7.0)	0 (0.0)	0 (0.0)	5 (9.1)	0 (0.0)	3 (8.3)	2 (10.5)	2 (11.1)	0 (0.0)	0 (0.0)
*Dose increased*	0 (0.0)	0 (0.0)	1 (2.3)	2 (10.5)	0 (0.0)	0 (0.0)	0 (0.0)	1 (2.8)	0 (0.0)	0 (0.0)	0 (0.0)	0 (0.0)

*p between groups >0.05. Length of cycles according to routine clinical practice: sunitinib 6 weeks; other treatments 4 weeks

**Fig. 2:**
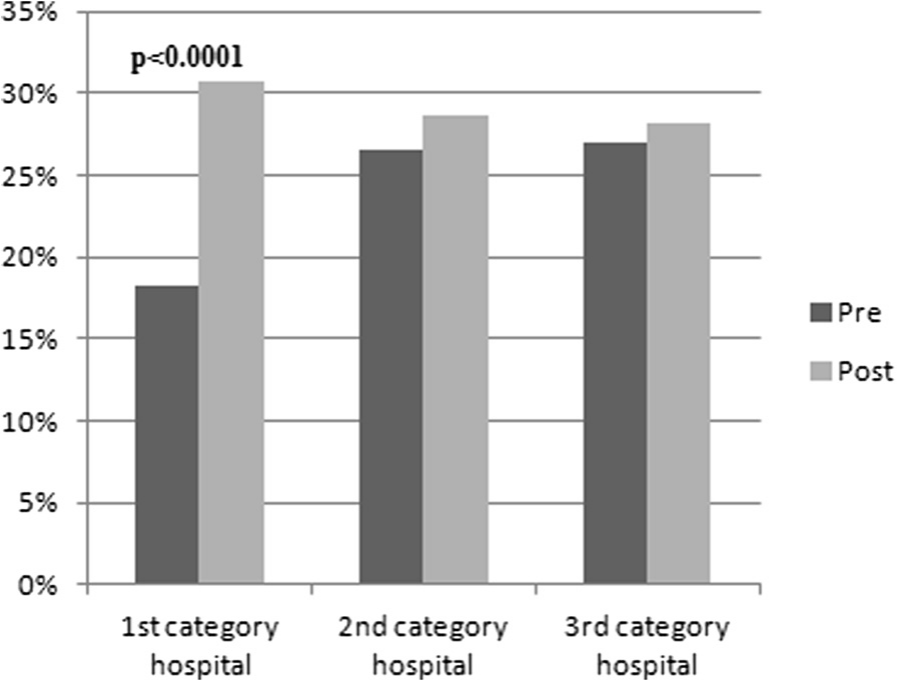
Adherence to SOGUG Guidelines according to hospital category defined as number of cases diagnosed with renal cancer per year (c/y): 1st category hospital (≥ 20 c/y); 2nd category hospital (11–19) c/y and 3rd category hospital (0 to 10 c/y)

Regarding the hospital category, a significantly greater adherence to the SOGUG recommendations was observed after the program was launched in those hospitals with a higher number of cases of renal cancer per year (18.2 vs. 30.7; *p* < 0001; Fig. 2). Hypertension (30 % vs. 56.0 % *p* < 0001) and hyperglycemia (60.5 % vs. 90.9 %; *p* < 0.001) were the adverse events that showed a significantly higher compliance with the guide after the implementation program in primary hospitals, and diarrhea (91.2 % vs. 96.5 %; *p* = 0.033) in secondary hospitals.

## Discussion

This study assessed the impact of the implementation and diffusion program of SOGUG guideline[15] for the management of targeted therapies in daily clinical practice.

Proper management of adverse effects ensures that patients receive optimal benefit from these newer therapies[9]. The aim of these Guidelines was to provide oncologists with a useful, easily handled tool in relation to strategies for prevention and management of AEs due to targeted agents. Overall, the present analysis showed a slight but significant improvement of adverse event management as a whole after the implementation of the SOGUG recommendations, and in particular with regard to hypertension, diarrhea and dyslipemia. Primary hospitals showed a meaningful increase in adherence to SOGUG Guidelines.

These recommendations reflect the consensus from an expert working group of medical oncologists. Nevertheless, clinical judgment based on the medical history and clinical status of the individual patient is actually what determines the appropriate management and the actions to be taken in response to side effects of targeted treatments. Strategies to evaluate the effectiveness and efficiency of guidelines dissemination and implementation have been also reported by different authors [16, 17]. Although the use of guidelines can improve clinical practice [18], both the context within which guidelines are delivered and the nature of target clinical behaviors may also influence their uptake [16]. In addition, clinical practice has proved remarkably resilient to recommendations for practice change embedded in clinical practice guidelines [19].

Although SOGUG recommendations were not complied with as a whole in nearly three-quarters of managed cycles, when adherence was analyzed by type of AE, appropriate management of some toxicities increased meaningfully. In particular, improvements were observed in hypertension and diarrhea management in sunitinib cycles and dyslipemia management with everolimus. Diarrhea is one of the most common toxicities, observed both with TKIs (50–60 %) and mTOR inhibitors (30 %) [10, 11]. Hypertension is, by itself, associated with organ damage, including left ventricular hypertrophy, congestive heart failure, coronary artery disease or myocardial infarction, and it is also one of the prime causes of proteinuria [20]. Optimal management of hypertension hypothetically reduces the appearance of long-term cardiovascular diseases. Hypertension occurs in 17–45 % of TKI-treated patients (40 % pazopanib, 30 % sunitinib, 4–11 % sorafenib) and bevacizumab (3–11 %) patients[10], but is rarely described with mTOR inhibitors[11]. Hypertension, in particular of grade 3, has been associated with a greater treatment response [21, 22] and may be considered an efficacy biomarker in patients treated with VEGF inhibitors [13, 20]. It presents early, within 3 to 4 weeks of treatment initiation [9, 23]. Hypertension should not be a reason for dose reduction nor treatment interruption as it can be safely managed with adequate treatment. Metabolic changes as hyperglycemia (26–57 %) or dyslipemia (52–77 %) are associated mainly with mTOR inhibitors [11].

SOGUG Guideline recommended performing at baseline and during therapy several tests that permit prevention and early detection of adverse events such as fatal hepatic failure, pneumonitis, hypertension or hyperglycemia, among others. Similar recommendations have been published by several authors [9–11, 20]. In this study, the most frequent reason for non-compliance with the Guidelines was failure to perform tests. The majority of laboratory abnormalities do not require intervention in most cases [11]. It is often difficult to differentiate between treatment-induced and disease-induced changes in some metabolic or laboratory parameters [11]. On the other hand, at RCC onset, elderly patients often suffer from some chronic diseases such as hypertension or dyslipemia, which requires treatment and monitoring to be conducted in the primary care setting. Based on these considerations, such tests were probably performed though not recorded in the medical history because their outcomes either did not have any notable significance or were performed by primary care physicians. Even so, one of the aims pursued with the implementation of the Guidelines was to make oncologists aware of the importance of conducting such tests and their monitoring.

The maximum benefit from antiangiogenic drugs is obtained in patients who can stay on therapy continuously over a prolonged period of time. Continuous therapy is possible only if the associated adverse events are effectively managed [20]. After SOGUG Guidelines implementation, we expected a significant decrease in dose reduction and temporary or final interruptions of treatment, but this was not observed. Treatment modifications rates were lower than those observed in other observational studies performed in the real-world clinical setting [24, 25]. The percentages of dose reductions/dose interruptions in the present study are lower than those reported from sunitinib’s pivotal [26] and SWITCH [27] trials. But in the range of that was shown in the EFFECT [28] study where 11 % of the patients treated with treatment schedule *4 weeks of treatment/2 weeks off*, needed treatment interruption due to adverse events. It is possible that in our study the use of non-standard treatment schedules, not permitted in clinical trials, may have contributed to maintain the doses without the need for dose reductions or interruptions during treatment. In addition, this is a cross-sectional study in which the treatment is analyzed in one period of time compared with to another period of time, therefore the data collected about patient’s exposure to the drug is less than in a clinical trial*.*

Methodological limitations need to be taken into consideration in this study. Firstly this study evaluated the Spanish Guidelines which limits the applicability in other countries. In addition, the outcomes may not reflect the complexity of the Guidelines. Non-compliance criteria were simplified for the purpose of making data collection feasible. Only the management of the most representative AEs for every treatment was recorded, which suggests the possibility of measurement bias. Secondly, the lack of patients before implementation Guidelines in the pazopanib treatment group and the small sample size of the bevacizumab group did not allow changes in outcomes to be detected in 61 of the 407 patients included.

## Conclusion

Slight but significant improvements in adverse event management in compliance with SOGUG recommendations were detected following their dissemination and implementation; in particular in hypertension, diarrhea and dyslipemia. Educational programs focused on the implementation of clinical guidelines can impact on the management of adverse events. However, room for improvement in the management of adverse events due to targeted agents still remains and this could be the focus for further programs in this direction. SOGUG Guidelines are already being updated to make them more accurate and precise in order to be really useful for management of AEs.
